# Protein Turnover in Aging and Longevity

**DOI:** 10.1002/pmic.201700108

**Published:** 2018-03-25

**Authors:** Nathan Basisty, Jesse G. Meyer, Birgit Schilling

**Affiliations:** ^1^ The Buck Institute for Research on Aging Novato CA USA

**Keywords:** aging, mass spectrometry, protein turnover, proteostasis, stable isotope labeling

## Abstract

Progressive loss of proteostasis is a hallmark of aging that is marked by declines in various components of proteostasis machinery, including: autophagy, ubiquitin‐mediated degradation, protein synthesis, and others. While declines in proteostasis have historically been observed as changes in these processes, or as bulk changes in the proteome, recent advances in proteomic methodologies have enabled the comprehensive measurement of turnover directly at the level of individual proteins in vivo. These methods, which utilize a combination of stable‐isotope labeling, mass spectrometry, and specialized software analysis, have now been applied to various studies of aging and longevity. Here we review the role of proteostasis in aging and longevity, with a focus on the proteomic methods available to conduct protein turnover in aging models and the insights these studies have provided thus far.

## 1 Introduction

Protein homeostasis (proteostasis) is the maintenance of a functional equilibrium between protein synthesis, fidelity, folding, localization, modification, and degradation. The ability to maintain and fine‐tune this equilibrium in response to internal and external cues is essential for cellular and organismal health. Loss of proteostasis is a “hallmark” of aging^1^, ^2^ that manifests at the cellular level in a number of ways, such as protein aggregation,^3^ unfolding,^4^ oxidative damage,^5^, ^6^ post‐translational modification,^7^, ^8^, ^9^, ^10^ and altered rates of protein turnover.^11^, ^12^ The importance of proteostasis is illustrated by many studies indicating that age‐related diseases and conditions are associated with the inability of the cell to maintain healthy proteins or eliminate defective proteins,^13^ as observed in neurodegenerative diseases,^14^ cardiac dysfunction,^15^, ^16^ cataracts,^17^ and sarcopenia.^18^

Interventions that extend healthspan and lifespan in various animal models have also suggested an important role of protein homeostasis in health and aging. While dysfunction of protein quality control mechanisms is a hallmark of aging, improvement of these mechanisms is associated with longevity and health. For example, defects in proteostasis have been alleviated in longevity models utilizing overexpression of mitochondrial‐targeted catalase,^19^, ^20^, ^21^, ^22^ calorie restriction (CR),^23^ reduced IGF1 signaling,^18^, ^24^, ^25^ and rapamycin (RM) treatment.^26^, ^27^ CR and rapamycin both inhibit the mechanistic target of rapamycin (mTOR) protein, which mediates protein translation and degradation rates in response to nutrient availability, increases lifespan and healthspan in a variety of animal models.^13^, ^14^, ^24^, ^28^

Changes in proteostasis can be quantified in several ways, often by observing changes in markers of synthesis and degradation machinery, protein quality, or protein aggregation. Only recently, sophisticated mass spectrometry and data analysis pipelines have enabled the comprehensive measurement of protein turnover rates with unmatched granularity and throughput. Proteins can only be synthesized by the ribosome, but they can be degraded by various means. Most proteins are degraded in the lysosomal pathway, largely through bulk degradation, or targeted specifically for degradation by the ubiquitin‐proteasome system (UPS).

## 2 Autophagy

Autophagy is one of the primary protein degradation systems of the cell, which utilizes the lysosomal pathway for degradation.^28^ There are three major ways by which proteins can be delivered to a lysosome for degradation: macroautophagy, microautophagy, and chaperone‐mediated autophagy. For detailed descriptions thereof readers are referred to several reviews.^13^, ^28^, ^29^ In the context of proteostasis, autophagy clears damaged proteins, insoluble protein inclusions, and abnormal organelles, all of which are hallmarks of aged and dysfunctional tissues. Loss of autophagy results in accumulation of damaged organelles and proteins.^30^, ^31^, ^32^

Autophagy components are required for the longevity conferred by several interventions: CR, mTOR inhibition, IGF‐1 inhibition, as well as other longevity‐associated pathways.^33^ It is less clear if autophagy activation is sufficient to extend lifespan because altering autophagy with complete specificity is not possible. For example, overexpression of ATG5, a protein required for autophagosome formation, extends lifespan in mice,^34^ but ATG5 also has pro‐apoptotic functions that may also contribute to longevity,^35^ and that may also contribute to longevity by protecting against cancer, particularly in cancer‐prone laboratory mouse strains.^36^ Various studies have reported longevity following interventions which inhibit mTOR, a known autophagy mediator, offering further evidence that autophagy plays a central role in aging.^28^, ^33^, ^37^

To connect protein synthesis and degradation, Mathis et al. recently examined the turnover rates of more than 1000 proteins and ribosomal RNA (rRNA) in mouse liver tissue from normal and CR animals.^38^ CR neither altered the average turnover rate for over 1000 measured proteins nor did it alter rRNA turnover. By comparing the rate of rRNA turnover with the rate of turnover for 71/80 integral ribosomal proteins (r‐proteins), they found that ≈80% of the r‐protein turnover rates were statistically indistinguishable from the rRNA turnover rate, suggesting that most ribosome synthesis and degradation occurs to the assembled unit, termed ribophagy. CR increased the rate of ribophagy from 10% per day to 11% per day. Notably, a few r‐proteins had higher turnover rates than the bulk ribophagy rate in both normal and CR animals, and these proteins are structurally present at the interface of the small and large ribosomal subunits. More experiments are needed to understand the functional importance of both altered bulk ribophagy, and the higher turnover rate of specific subunits relative to the whole complex. For example, would overexpression or knockdown of the fast‐turnover subunits have biochemical or functional effects in vivo? And what factors or conditions mediate the bulk turnover of ribosome?

## 3 Mitophagy

Mitochondrial‐specific autophagy, or “mitophagy,” is a process by which defective mitochondria are turned over through the lysosomal pathway. Knocking down components of the autophagosome or lysosomal pathway strongly diminish mitochondrial function,^29^, ^39^, ^40^ demonstrating that it plays a key role in mitochondrial maintenance and homeostasis. Two well characterized regulators of mitophagy are PINK1 and parkin.^41^, ^42^ PINK1, aka phosphatase and tensin (PTEN) homologue‐induced kinase 1, is a mitochondria‐targeted serine/threonine kinase which serves to protect the cell from mitochondrial dysfunction and apoptosis.^43^ PINK1 mutations are the most common cause of recessive familial Parkinsonism in humans.^44^ In addition, PINK1 KO mice exhibits severe deficiencies in mitochondrial homeostasis accompanied by morphological changes in the mitochondrial network, increased ROS, and susceptibility to heat shock.^43^ Together this evidence suggests defective mitophagy plays an important role in Parkinson's disease as well as overall mitochondrial quality in healthy cells and suggests an important role in aging. Mitophagy is also required for the lifespan extension conferred by both mitochondrial stress and reduced IGF‐1 signaling.^45^ In *Drosophila*, overexpression of parkin, a protein required for mitophagy, is sufficient to extend lifespan.^46^ Further details on the role of mitophagy in aging and longevity is reviewed elsewhere.^47^

## 4 Ubiquitin‐Mediated Protein Degradation

The UPS is the primary non‐lysosomal protein degradation pathway. Compared to autophagy, which often degrades proteins in bulk, the UPS utilizes a sophisticated array of mechanisms to target individual proteins and does so with spatial and temporal precision. The UPS is also active in all regions of the cell, and targets proteins localized within organelles. For most proteins, degradation through this pathway is characterized by two major steps: recognition and “tagging” of a protein for elimination via poly‐ubiquitination, and translocation to the proteasome for degradation.^29^ The details of this complex system are detailed in various reviews.^13^, ^28^, ^29^, ^48^

Like autophagy, the UPS is essential for maintaining proteostasis. Inhibiting or deleting its components often leads to toxicity, severely altered cellular phenotypes, and cellular death.^48^, ^49^ Almost immediately following inhibition of the proteasome, accumulation of protein inclusions can be observed in cultured cells. Interestingly these resemble the inclusions described in many age‐related neurodegenerative diseases.^48^, ^49^, ^50^, ^51^ Genetic depletion of proteasome subunits in the brains of mice has been shown to induce a neurodegenerative phenotype,^50^ suggesting functional UPS is required to prevent neurodegenerative diseases.

The UPS appears to influence longevity through specific degradation of proteins in longevity pathways, rather than bulk changes in degradation. The ubiquitin ligase RLE‐1, for example, selectively poly‐ubiquitinates daf‐16, a key component in the insulin/IGF pathway in worms, leading to its degradation by the proteasome.^52^ As a result, inhibition of RLE‐1 extends lifespan in *Caenorhabditis elegans*. Similarly, overexpression of parkin‐1 a ubiquitin ligase involved in familial Parkinson's disease, extends lifespan of flies.^46^

Overall, the extent to which UPS is involved in aging is not understood. Proteasome function declines with age but is restored in long‐lived animals under CR.^13^, ^28^ Autophagy and the UPS must work in harmony to maintain proteostasis, and alteration of either process inevitably changes in both systems. For example, autophagy and the UPS interact in host‐cell autonomous immunity, where the autophagic destruction of invading pathogens relies on the extensive ubiquitination of pathogen components distinguishing pathogens as “non‐self.”^53^

## 5 Protein Synthesis

The idea that reduced protein synthesis may promote longevity was popularized soon after it was discovered that calorie restriction and rapamycin treatment, interventions known to reduce protein translation through inhibition of mTOR, extend lifespan in organisms across the evolutionary spectrum. In mammals, mTOR regulates protein translation primarily through two substrates, ribosomal protein S6 kinase (S6K) and eukaryotic translation initiation factor 4E‐binding protein 1 (4E‐BP1). It has now become clear that reduction of translation via reduction of mTOR, S6K, 4E‐BP1, or components of translational machinery has a clear association with longevity across evolutionary diverse organisms, including yeast,^54^ worms,^55^, ^56^ flies,^57^, ^58^ and mice.^59^ While it is not entirely clear how reduced protein synthesis promotes longevity, several possibilities have been proposed. One possibility is that slower translation reduces the load on other proteostasis machinery, allowing protein folding and degradation processes to reduce the burden of misfolded, damaged, and aggregated proteins.^60^ Reducing mRNA translation, which consumes about 50% of total cellular energy,^61^ could allow energy to be diverted to cellular maintenance and repair processes. Slower translational elongation has also been shown to improve protein translational fidelity,^62^ thereby resulting in higher quality proteins and possibly promoting health. The importance of translational fidelity in aging is supported by a recent comparative study across 17 rodent species with diverse lifespans, which reported a strong negative correlation between maximum lifespan and amino acid misincorporation, suggesting that translational fidelity coevolved with longevity.^63^

Collectively, studies on protein synthesis, quality control, and degradation pathways strongly suggest that maintenance of proteostasis is essential for health and longevity. While changes in turnover machinery with age are well documented, robustly measuring the turnover of proteins targeted by these mechanisms has only recently become possible.

## 6 Methods for Measuring Protein Turnover

Protein turnover has been of interest for the community for a long time and approaches have been previously reviewed.^64^, ^65^ Here, we provide a brief survey of methods with focus on the factors that influence the choice of strategy. Although classical approaches to measure protein turnover have used radioactive pulse chase experiments, methods using stable‐isotope labels and mass spectrometry have far greater throughput and accuracy. Importantly, proteomic workflows to measure protein turnover can provide results for thousands of individual proteins rather than “bulk” up or down regulation of protein half‐lives. We also explore a few data analysis options for use with each general strategy.

**Figure 1:**
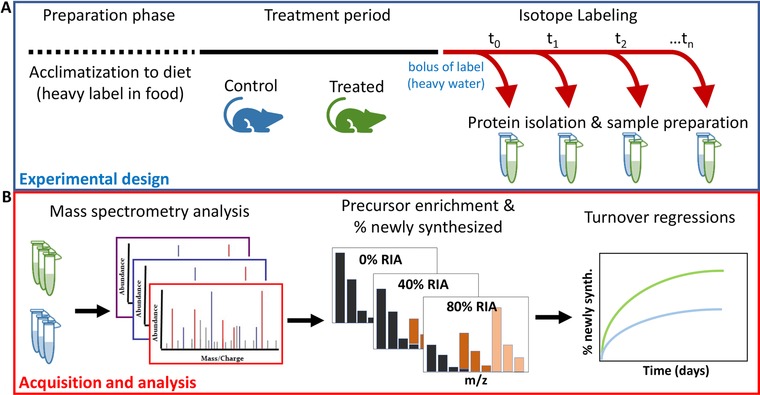
General workflow for measurement of in vivo protein turnover rates in rodents using mass spectrometry. A) The use of heavy‐labeled amino acids generally requires a synthetic diet of a similar composition to regular chow, and it is important to acclimate animals to the non‐labeled synthetic diet for a few weeks prior to the start of the experiment. Mouse treatments, if used, are usually administered prior to supplementation of heavy label. For heavy‐water labeling, an initial bolus of labeled water is injected at the start of the labeling period, followed by supplementation of a lower percentage of deuterium in the drinking water. Otherwise, label is supplemented in the chow during this period. Tissues from all treatment groups are then collected at several time points, usually on the order of days to weeks, and processed for mass spectrometry analysis. B) For comprehensive survey of turnover, samples are usually analyzed by mass spectrometry using data‐dependent acquisition. An analysis of peptide isotopomer peaks is then conducted using specialized software (e.g. Topograph) to determine the enrichment of label in the precursor pool and the percentage of each protein that is newly synthesized. For each protein, a regression of the fraction that is newly synthesized is then performed to determine its rate of turnover.

Typically, for a proteomics‐based, continuous labeling protein turnover experiment, organisms are fed a synthetic, stable‐isotope‐enriched diet (Figure 1A). Cells or animals will be kept on this synthetic diet until defined time points are reached and then harvested or sacrificed. Mass spectrometric analysis of tissues from each time point is typically performed in data‐dependent acquisition mode (DDA). During the data analysis phase (Figure 1B), specialized software (discussed below) is used to perform an analysis of relative intensity of peptide isotopomer peaks and determine the fraction of each protein that is newly synthesized at each time point. The rate of incorporation of newly synthesized proteins over time reflects rates of protein turnover.

The choice of heavy isotope depends primarily on the biological model under investigation and influences the way mass spectrometry data is collected and analyzed. In yeast, metabolic labeling is typically achieved by growing yeast in media containing ^15^N labeled ammonium sulfate as the sole nitrogen source or on labeled amino acids.^66^ To avoid the utilization of unlabeled amino acid generated from alternate metabolic pathways, a yeast strain that is auxotrophic for the labeled amino acid should be used. *Drosophila melanogaster* and *C. elegans* can be labeled by ^15^N or amino acids by feeding of fully labeled yeast or *Escherichia coli*, respectively.^66^ Metabolic labeling of rodents can be done by supplementing the diet with a heavy amino acid or ^15^N enriched algae (Spirulina).^66^ Low‐level D_2_O labeling, delivered by an intraperitoneal injection of nearly pure D_2_O followed by continuous labeling by supplementation in drinking water, is also gaining popularity.^67^

Given the options of labels and incorporation routes, it is useful to critically compare the strengths and weaknesses of each of the three classes of label incorporation regarding cost, data analysis. While labeling via ^15^N‐enriched diets and D_2_O is relatively inexpensive, the resulting complex isotopic distributions of protein and peptide envelopes pose some data processing challenges and can be difficult to resolve. Labeling with heavy‐oxygen (H_2_^18^O) in place of hydrogen (^2^H_2_O) reduces the complexity due to incorporation of multiple ^2^H precursors into several amino acids,^68^ but this form of heavy water is significantly more expensive. Still, there are tools to analyze such complicated data.^69^ Heavy‐labeled amino acids result in larger and more easily resolved mass shifts and produce less complex isotopic distributions. However, the cost of heavy amino acids can be significantly higher than ^15^N or deuterium labeling compounds. When the label is delivered via diet in rodents, it is important to acclimate animals to the specialized diet without label to prevent disruptions in feeding and weight gain while label is administered. Essential amino acids of high frequency are preferred, such as ^2^H_3_‐Leu, ^2^H_8_‐Val, as doubly labeled peptides are required to determine label enrichment in the amino acid precursor pool. ^13^C_6_‐Lys may also be used, for which a Lys‐C digestion would be preferable to trypsin. D_2_O labeling, while currently not highly represented in aging studies, is broadly gaining popularity among turnover studies, and will likely be seen with increasing frequency in the aging field. D_2_O that does not require an acclimatization period has the considerable advantage that the precursor enrichment can be measured by measuring the enrichment of water in blood, thus avoiding having to algorithmically determine the precursor enrichment.

There are several options available to analyze the mass spectrometry–measured isotope ratios of peptides, which largely depend on the type of stable‐isotope labeling used. Topograph can be used with any type of metabolic label, including amino acids or metabolic intermediates, to determine turnover rates corrected to precursor enrichment.^70^ Guan et al. have described a modular analysis pipeline to determine turnover rates for ^15^N metabolic labeling.^71^ SILACtor performs turnover analysis for SILAC‐based labeling studies.^72^ A recently released DeuteRater software package,^73^ or the ProTurn java application,^74^ can be used to calculate protein turnover following heavy water labeling. A package written in R, *ProteinTurnover*, can be used to analyze turnover data from inorganic labeling experiments.^69^ There are several other examples of freely available tools and strategies for the analysis of mass spectrometric protein turnover data, including Gaussian process modeling^75^ and compartment modeling.^76^

Numerous studies have reported proteome‐wide turnover rates using the isotopic labeling methods described above with animal models ranging from unicellular organisms to humans in various tissues and biological contexts. Here we will focus only on a narrower subset of studies in the context of aging and aging interventions, however, for further detail on turnover studies beyond the scope of aging readers are referred to several reviews.^64^, ^65^

## 7 Protein Turnover in Aging Invertebrates

Several genetic and environmental interventions that extend lifespan are now known to be conserved across the evolutionary spectrum.^26^, ^77^, ^78^ The most highly studied longevity pathways in mammals, including insulin/insulin‐like signaling (IIS), FOXO, and TOR pathways, were first discovered and studied in simpler organisms such as *Saccharomyces cerevisiae* (yeast), *C. elegans* (nematodes), and *D. melanogaster* (fruit flies).^26^, ^77^, ^78^ Due to the conservation of aging genes, short lifespans, and genetic amenability, invertebrates are widely used as models in aging research. Interestingly, conserved aging pathways and interventions are also well known to alter global cellular proteostasis in response to environmental nutrient cues, and therefore studies of proteome homeostasis in long‐lived mutants may shed light on the specific mechanisms by these pathways which contribute to longevity.

A few proteomic studies have examined protein turnover in aging invertebrates. In budding yeast, lifespan can be measured by the number of cell divisions that a single yeast cell can undergo prior to senescence, termed replicative lifespan. A major determinant of yeast replicative lifespan is asymmetric cell division, the process whereby cellular components are asymmetrically distributed between a mother cell and daughter cell during division.^79^ Thayer et al. utilized heavy amino acid labeling coupled with mass spectrometry to identify ≈136 long‐lived proteins or protein fragments that were asymmetrically retained within the yeast mother cell throughout multiple cell divisions.^80^ In a separate study, Yang et al. used a high throughput flow cytometry‐based approach to identify 74 mother cell‐enriched proteins.^81^ Deletion of the genes corresponding to these proteins was sixfold more likely to extend lifespan than genes chosen at random.^81^ Together, these studies suggest that asymmetric retention and accumulation of proteins within mother cells may be a contributor to yeast replicative aging. This mechanism of replicative aging may also extend to more complex multicellular organisms, however, confirming this possibility would require further study in these models.

The nematode *C. elegans*, in contrast to yeast, undergoes no cell division in its somatic tissue and is composed of 959 cells throughout its adult lifespan^82^ and is therefore not ideal for modeling aging in proliferating cell types. However, nematodes are a useful model of chronological aging in post‐mitotic cell types such as neurons and muscles. A handful of studies have comprehensively examined in vivo proteome turnover in *C. elegans* aging and longevity^83^, ^84^, ^85^, ^86^ (Table 1). All studies reported a decrease in global proteome turnover in aged adult nematodes, especially proteins in microtubules, vitellogenins, translation (e.g., ribosomes), and mitochondria. This decline is reversed in long‐lived strains, such as the insulin‐like growth factor receptor (daf‐2 –/–) mutant (Table 1).^83^, ^86^ Interestingly, the opposite pattern is observed in young adults, where daf‐2 mutants have slower global protein turnover compared with controls.^83^, ^86^

**Table 1:** Studies of in vivo protein turnover with age or following an aging‐intervention

Organism	Tissue	Ages	Change in global turnover with age	Top protein and pathway changes (aging)	Intervention	Change in global turnover with intervention	Top protein and pathway changes (intervention)	Ref.
*C. elegans*	Whole organism	Days 1–7 of adulthood	Decrease	**Decreased—**microtubili, vitellogenins, cytoskeletal proteins, ribosomal proteins, lysosomal proteins, mitochondrial proteins, glycolysis, TCA cycle **increased—**SOD‐1, PRDX‐3	N/A	N/A	N/A	83
*C. elegans*	Whole organism	L4 to day 5 of adulthood	Decrease	N/A	daf‐2 mutant (e1370)	Increase	**Decreased—**N/A **increased—**lipid transport, nutrient reservoir activity, P granule	82
*C. elegans*	Whole organism	Days 2–14 of adulthood	Decrease	N/A	daf‐2 mutant (e1370)	Increase	N/A	85
*C. elegans*	Whole organism	Days 1–10 of adulthood	Decrease	N/A	N/A	N/A	N/A	84
Mouse	EDL/soleus muscle mitochondria	Young: 5–8 months Old: 27–29 months	Decrease	**Decreased—**glycolysis **increased—**mitochondrial function, oxidative phosphorylation, calcium signaling, TCA cycle	N/A	N/A	N/A	90
Mouse	Heart	Young: 4 months Old: 26 months	No change	**Decreased—**mitochondrial function, oxidative phosphorylation, calcium signaling, TCA cycle **increased—**protein ubiquitination	Calorie restriction (CR) and RP	Decrease	**Decreased—**N/A **increased in CR and RP—**mitochondrial function, oxidative phosphorylation, calcium signaling, TCA cycle, protein ubiquitination	11
Mouse	Liver	Young: 3 months Old: 25 months	Increase	**Decreased—**protein translation, ribosomal proteins **increased—**mitochondrial function, oxidative phosphorylation, BCAA, fatty acid oxidation, glycolysis	Calorie restriction (CR) and RP	Decrease	**Decreased in CR—**mitochondrial function, oxidative phosphorylation, BCAA, fatty acid oxidation, glycolysis, acute phase response, primary immunodeficiency signaling, glutaryl‐CoA degradation **increased in CR—**protein translation, ribosomal proteins **Decreased in RP—**primary immunodeficiency signaling, protein translation, ribosomal proteins **increased in RP—**BCAA, glutaryl‐CoA degradation	12
Mouse	Liver	18 months	N/A		CR	Decrease	**Decreased in CR—**mitochondrial proteins **increased in CR—**N/A	92
Mouse	Heart/liver	Young: 3–4 months Old: 18–21 months	Increase	**Decreased—**N/A **increased—**mitochondrial function, LPS/IL1‐mediated RXR function, fatty acid oxidation, xenobiotic metabolism, aryl hydrocarbon receptor signaling, BCAA, TCA cycle, actin cytoskeleton, nor/adrenaline degradation, ethanol degradation	Overexpression of mitochondrial‐targeted catalase (mCAT +/+)	Decrease	**Decreased—**mitochondrial function, LPS/IL1‐mediated RXR function, fatty acid oxidation, xenobiotic metabolism, aryl hydrocarbon receptor signaling, BCAA, TCA cycle, actin cytoskeleton, nor/adrenaline degradation, ethanol degradation **increased—**N/A	91
Mouse	Liver	5–7 months (Snell) 8 months (Rapa) 18 months (CR)	N/A		Snell dwarf (Pit1‐/‐), CR, rapamycin (RP)	Decrease	**Decreased in Snell dwarf—**protein processing in the ER **increased in Snell dwarf—**glutathione S‐transferases **Decreased in CR—**Protein processing in the ER **increased in CR—**N/A **Decreased in RP—**protein processing in the ER **increased in RP—**glutathione S‐transferases	93

## 8 Protein Turnover in Aging Mammals

Several studies have measured global proteome turnover changes in rodent aging. In a survey of extremely long‐lived proteins in rats, Toyama et al. performed an ^15^N pulse‐chase experiment in brain, liver, and lens tissue that was collected at several time points up to 1 year.^87^, ^88^ This survey found that the extremely long‐lived proteome consisted mostly of nuclear pore proteins, histones, collagens, and, surprisingly, a few enzymes: Enpp6, Sirt2, and Asrgl. Among proteins in the structural scaffold of the nuclear pore complex (NPC), 25% were found to persist for over a year in neurons. Interestingly, all NPC proteins maintained constant synthesis rates with age, but the long‐lived components were the only ones that decreased in abundance. Since the subcomplexes of the NPC have variable rates of turnover, a decline in the abundance of long‐lived components with age may be explained by difficulty in disassembling and reassembling of the structural subcomponents of the entire NPC complex. The apparent inability for neurons to replace structural NPC components may underlie the decline of nuclear membrane integrity and leaking of cytoplasmic molecules observed in the nucleus of aging post‐mitotic cells.^89^ Exceptionally long‐lived proteins in general are also inherently vulnerable to various modifications, unfolding, and damage. The accumulation of these changes may also underlie an age‐related loss of protein function and increase in protein aggregation. This is likely the case with crystallin proteins, identified among exceptionally long‐lived proteins in the lens of the eye,^87^ which are known to accumulate several modifications over time and eventually form insoluble aggregates during the pathogenesis of age‐related cataracts.^90^

In general, proteomic studies examining global changes in turnover across the mouse proteome have reported little or no overall change with age. Kruze et al. examined protein turnover in two skeletal muscles, the extensor digitorum longus and the soleus, in mice and found that, in general, turnover was not significantly changed in with age, although there was a modest reduction in the turnover of mitochondrial proteins.^91^ The Rabinovitch group has also conducted several studies of proteome turnover in the hearts and livers of normally aged mice as well as in mice given several well‐known aging interventions including: calorie restriction, rapamycin treatment, and overexpression of mitochondrial‐targeted catalase.^11^, ^12^, ^92^ Across these studies, global turnover of proteins with age was significantly changed in many individual proteins, although when taken as a bulk global measurement, proteome turnover was either slightly increased or not significantly altered (Table 1). Among individual proteins, changes in turnover from aging in both heart and liver were enriched into several common pathways (Table 1) such as mitochondrial dysfunction (mostly composed of electron transport chain proteins), branched chain amino acid metabolism, actin cytoskeleton, oxidative stress response, ethanol degradation, and aryl hydrocarbon receptor signaling.^11^, ^12^, ^92^

More striking than age‐related changes in proteome turnover are the changes induced by interventions which slow aging. Across numerous studies, aging interventions have the effect of decreasing bulk turnover of the proteome in aged mice (Table 1). This observation was consistent across several longevity models: calorie restriction,^11^, ^12^, ^93^, ^94^ rapamycin treatment,^11^, ^12^, ^93^, ^94^ overexpression of mitochondrial‐targeted catalase,^92^ and Pit1 knockdown (Snell dwarf mice).^94^ The strongest effects were seen in pathways that were most highly altered with age, including mitochondrial dysfunction, TCA cycle, fatty acid oxidation, and BCAA metabolism, and were generally of much greater magnitude than aging itself. Dai et al. also demonstrated, by ex vivo substrate utilization and metabolomics, that these changes were accompanied by a shift away from mitochondrial metabolism and fatty acid utilization toward glycolytic metabolism with age, well‐documented features of both aging and cancer.^95^ Importantly, calorie restriction and rapamycin treatment reversed the age‐related changes in the turnover of these metabolic proteins and restored youthful metabolic function, suggesting that age‐related declines of mitochondrial metabolism may be reversed by targeting the quality control and maintenance machinery of metabolism at the protein level.

In the cases of calorie restriction, rapamycin treatment, and overexpression of mitochondrial‐targeted catalase, longer proteome turnover in aged animals was accompanied by improved proteome quality, as measured by reductions in protein carbonylation and poly‐ubiquitination.^11^, ^12^, ^92^ A more recent study out of the Rabinovitch group examined protein turnover among ubiquitinated proteins in aging, and found that a striking proportion of ubiquitinated proteins were not turning over in aged animals.^96^ This effect was not present in young animals and was alleviated or reversed in calorie restricted or rapamycin‐treated mice. These observations raise the question of whether enhanced protein quality and reduced protein turnover is a common underlying longevity mechanism in these and perhaps other interventions. This has been proposed by Thompson et al. in a study of protein half‐lives across multiple longevity interventions.^94^ It was also proposed in this study that reduced turnover may serve as a biomarker for interventions that delay aging in mammals. While the reason for this is not clear, it is possible that slower rates of protein turnover are indicative of a resilient state of proteome homeostasis which requires less maintenance. Dietary restriction, for example, triggers an adaptive shift of cellular resources toward cellular and somatic maintenance, including improved proteome quality and stability.^97^ In this state, in which the proteome is buffered by chaperones and cellular antioxidants, proteostasis may be less susceptible to perturbation by cellular stressors and damage, thereby requiring less protein turnover for maintenance.

In humans, several studies have examined protein turnover in sarcopenia or age‐related skeletal muscle atrophy. Muscle strength and mass is typically maintained until middle age, after which accelerated losses occur in both.^98^ Early isotopic labeling studies in humans, usually by continuous infusion of l‐[1‐^13^C]leucine and collection of skeletal muscle biopsies, established that aging led to a reduction in the synthesis rates in the skeletal muscle, in mixed muscle protein, mitochondrial proteins, and myosin heavy chain, which may underlie age‐related decline in muscle mass.^99^, ^100^, ^101^, ^102^ Further studies have found that both resistance and aerobic exercise training increase muscle protein synthesis and improve muscle function irrespective of age and may help counteract some of the effects of aging.^99^, ^103^, ^104^

## 9 Challenges Remaining for Measuring Protein Turnover

The labeling strategies for animals for protein turnover studies face various challenges and typically full heavy labeling of peptide precursor ions is difficult to achieve.^105^ Thus, labeling intervals in excess of 30 days may be needed to guarantee label incorporation for proteins with slow turnover. The time needed for efficient protein label incorporation covering the dynamic range for proteins with both fast and slow protein turnover is directly related to labeling period and metabolic stable‐isotope labeling costs. While most proteins can be measured with labeling period on the order of weeks, this may not be sufficient time to observe label incorporation in a small number of very long‐lived proteins. Conversely, short‐lived proteins may be missed if tissue collection is not performed early enough in the labeling period. In addition, protein turnover is different for individual proteins depending on tissue type or subcellular localization. For example, Claydon et al. reported that the median rate of degradation of muscle protein is considerably lower than liver or kidney, while heart protein turnover showed generally intermediate turnover rates.^105^ Subcellular specificity of protein turnover dynamics was investigated by Larance et al. who have assessed protein turnover and specifically identified rapidly degrading proteins upon cycloheximide treatment and annotated proteins for stability and subcellular distribution.^106^

In addition, the tissue, cell‐type, and even cell compartment may also present a challenge in determining in vivo turnover with stable‐isotope labeling due to potential differences in precursor pool enrichments. While current methods can calculate turnover based on models of one or more pools, no tools exist to measure these potentially distinct pools separately. This presumably poses more of a problem in tissues that are highly heterogeneous in cell‐type or in model organisms like *C. elegans*, in which case turnover is calculated (usually based on a single pool model) in a lysate composed of whole tissue or organism.

## 10 New Innovations and Future Methods for Measuring Turnover

### 10.1 Isobaric Tagging

Most approaches for quantification of protein turnover use MS1 (precursor ion) quantification to calculate protein half‐lives, where each time point must be analyzed by a separate mass spectrometry run. A new workflow has recently been developed to improve quantitative accuracy and achieve a higher multiplexing for protein turnover studies. Turnover experiments still undergo continuous enrichment using traditional metabolic labeling, such as SILAC, but each time point is chemically tagged using tandem mass tags (TMT) or isobaric tags for relative and absolute quantification (iTRAQ). Subsequently, the samples labeled with a four‐plex, ten‐plex, or similar isobaric tag, are mixed before MS analysis. Fragmentation of the heavy or light metabolic label then reveals the reporter ion clusters that reflect rates of newly synthesized or pre‐existing protein, respectively. This strategy was first demonstrated by Jayapal et al. in 2010 to estimate 115 protein turnover rates in *Streptomyces coelicolor* cultures undergoing transition from exponential growth to stationary phase.^107^ Cultures were grown in labeled medium and transferred to unlabeled medium at the switch to stationary phase. A secondary labeling step was performed with iTRAQ reagents at four different time points (four‐plex) to determine protein turnover rates. This dual labeling strategy enabled both peptide identification and quantification of turnover dynamics used from the MS/MS spectra. One related recent study by Welle et al. used a combination of ten‐plex TMT, SILAC, and MS3‐level reporter ion quantification for multiplexed turnover measurement of over 3000 proteins along ten time points.^108^ Although these multiplexing strategies for measuring protein turnover provide the same information as traditional MS1‐based strategies, these methods will find application in many scenarios where throughput is a priority.

### 10.2 Protein Turnover using MS2‐based Mass Spectrometric Approaches: Data‐Independent Acquisition

Protein turnover has traditionally been processing MS1‐based quantification from DDA analyses, which is best performed on high mass resolution instruments. However, Holman et al. recently assessed selected reaction monitoring‐mass spectrometry (SRM‐MS) for protein turnover measurement.^109^ In this latter study adult male mice were fed a stable‐isotope diet containing [^13^C_6_]lysine for varying amounts of time ranging from 0 to 30 days (11 time points), and MS1‐DDA analysis was performed in parallel to targeted SRM analysis. The authors reported that SRM outperformed MS1 in terms of sensitivity and selectivity of measurement, allowing more confident determination of protein turnover rates.^109^ Holman et al. pointed out that SRM acquisitions are well suited for focused studies measuring the turnover of tens of proteins and determining the dynamics of proteins complexes and complete metabolic pathways. The targeted SRM approach can take advantage of the higher selectivity and specificity of the SRM precursor/fragment ion pairs, and SRM workflows typically show very good assay sensitivity and dynamic range for this MS2‐based quantification strategy. Some limitations of a SRM‐protein turnover approach are that (i) SRM assays will have to be developed before data acquisition, and (ii) multiplexing is limited, however, modern retention time scheduling can significantly increase SRM multiplexing capabilities.^110^

To overcome SRM multiplexing limitations, the Schilling lab is in the process of implementing untargeted, comprehensive MS2‐based approaches such as data‐independent acquisition (DIA) for measuring protein turnover (unpublished data). DIA workflows (e.g., SWATH) are high‐throughput, unbiased, and have reduced interference of fragment ion signals from co‐eluting peptides compared to MS1‐based quantification from DDA.^111^, ^112^ Thus, DIA strategies should provide improved accuracies for protein turnover measurements and allow for large scale assessments of tissue proteostasis. Novel features will be added to the Topograph^70^ and Skyline^113^ software platforms with new capabilities to process DIA‐MS2 fragment ions for protein turnover. We have recently reported this concept and workflows of using SWATH MS2 data for quantification of SILAC‐like, stable‐isotope‐labeled peptides (initially demonstrated in the context of stoichiometry,^114^ which can be adapted for the protein turnover calculations [“DIA turnover”]. In recent years DIA or SWATH workflows have gained tremendous attention and have become more and more popular over the last few years as the method of choice for high‐throughput label‐free quantification.^111^, ^115^, ^116^, ^117^ Applying these new MS technologies to biological protein turnover projects (“DIA‐turnover”) will allow for highly accurate and high‐throughput proteostasis studies.

## 11 Concluding Remarks

Mass spectrometric workflows have elevated protein turnover studies to a level of unprecedented detail into dynamic changes of protein turnover for individual proteins and entire proteomes. In the few years these approaches have been applied to the study of aging and longevity, researchers have uncovered that the turnover of specific proteins and pathways are impacted more strongly by aging and aging interventions than others. Studies have also shown a striking correlation between reduced protein turnover and slower aging, suggesting that targeting proteostasis machinery to slow down turnover may be a promising approach to mitigate age‐related diseases.

**Abbreviations**
CR: calorie restriction
DDA: data‐dependent acquisition mode
DIA: data‐independent acquisition
NPC: nuclear pore complex
RP: rapamycin
rRNA: ribosomal RNA
SRM: selected reaction monitoring
UPS: ubiquitin‐proteasome system

## Conflict of Interest

The authors have declared no conflict of interest.
